# Supplementary material for: WordCommentsAnalyzer: A windows software tool for qualitative research
Source: F1000Res. 2018 Sep 4;7:536. Originally published 2018 May 3. [Version 2] doi: 10.12688/f1000research.14819.2 (PMC6137411; doi:10.12688/f1000research.14819.2)
Supplement: Supplementary file 3 [file f1000research-7-17614-s0002.tgz › b2a79496-4ada-47c4-9682-a99ce867836d.zip!/Eight Iranian Health Journals/The Journal of Toloo-e-Behdasht_5.docx]

© The Journal of Toloo-e-Behdasht. This work is licensed under CC BY 4.0 (https://creativecommons.org/licenses/by/4.0/).

Volume & Issue: دوره 16 - شماره 3

Title: اثرات باریکه الکترونی بر میزان اکسیداسیون چربی در سوسیس

http://tbj.ssu.ac.ir/article-1-1915-fa.html

مقدمه: تیمار پرتودهی یکی از بهترین روش‎ها برای افزایش عمر مفید گوشت بدون اثرات سوء مغذی و ویژگی‎های حسی است. اگرچه باریکه الکترونی امکان ایجاد تغییراتی در مواد غذایی را دارد، اما به عنوان آسان‎ترین روش پرتودهی شناخته شده است . باریکه الکترونی دوستدار محیط زیست، کم هزینه و یک جایگزین موثر غیر وقت‎گیر نسبت به دیگر روش‎های آلودگی‎زدایی است. اکسیداسیون چربی می‎تواند در گوشت پرتودهی شده تولید شود. هدف از این مطالعه ارزیابی میزان اکسیداسیون چربی در سوسیس‎های پرتودهی شده است. یافته‎های این مطالعه می‎تواند در کنترل، ارتقا ایمنی مواد غذایی و ویژگی‎های کیفیتی در صنایع غذایی اثر بخش باشد. روش بررسی: سوسیس از مرکز تولیدی تهیه شد، سوسیس چرخ شده به منظور آنالیز تیوباربوتیریک اسید مخلوط و به قسمت‎های 25 گرمی تقسیم شد. نمونه‎ها شامل یک گروه شاهد و چهار گروه تیمار بودند. سوسیس‎های بسته‎بندی شده در معرض دوز‎های 0، 1، 2، 3 و 5 کیلوگری قرار گرفته و در روز‎های مختلف 0، 5، 10 و 30 بررسی شدند. یافته‎ها: میزان تیوباربوتیریک اسید با گذر زمان افزایش یافت. در دوز‎های مختلف ارتباط معنی‎داری مشاهده شد (05/0>P)، اما بیشترین میزان تیوباربوتیریک اسید در دوز 3 کیلو‎گری مشاهده شد. نتیجه گیری: بکارگیری باریکه الکترونی در دوز‎های پایین تاثیر سوئی بر اکسیداسیون چربی در سوسیس ندارد. پرتودهی این محصولات با افزودن آنتی اکسیدان‎ها می‎تواند در کاهش یا پیشگیری اکسیداسیون چربی موثر باشد.

Title: کاهش میزان پراکسیداسیون لیپید ها (مالون دی آلدئید) در بیماران دیابتی نوع II با مصرف پودر گیاه کندل کوهی

http://tbj.ssu.ac.ir/article-1-738-fa.html

مقدمه: افزایش گلوکز خون یکی از مهمترین عوامل ایجاد کننده ی  استرس اکسیداتیو دربیماری دیابت و درنهایت ایجاد عوارض قلبی - عروقی است. هدف از مطالعه ی ما بررسی اثر پودر گیاه کندل کوهی بر میزان پراکسیداسیون لیپیدها دربیماران دیابتی بود. روش بررسی: این مطالعه به صورت کارآزمایی بالینی کنترل دار تصادفی  و دوسوکور، بین 150 نفر ازمبتلایان به دیابت نوع ۲ در مرکز تحقیقاتی درمانی دیابت یزد انجام شد.با تقسیم بندی بیماران در سه گروه به ترتیب، روزانه کپسول های 100 و 500 میلی گرمی پودر گیاه و دارونما داده شد. نمونه های خون قبل و بعد از مصرف پودر گیاه گرفته شد. پس از اندازه گیری شاخص ها، داده ها با نرم افزار SPSS  نسخه 16 و با سطح اطمینان 95%  و05/0 P<، مورد تجزیه و تحلیل قرار گرفتند. یافته ها: میانگین مالون دی آلدئید، قبل و بعد از  مصرف به  ترتیب  در گروه  دارونما :03/0 ± 59/0 و  03/0 ± 58/0 ، در گروه دوز100 میلی گرمی 07/0 ± 44/0 و 09/0 ± 41/0  و برای دوز 500 میلی گرمی 07/0 ± 45/0 و 04/0 ± 26/0  میکرومولار بدست آمد. در گروه دوز 500 میلی گرمی، شاخص های چربی خون و قند خون ناشتا دارای کاهش بسیارمعنی داری بعد از مصرف پودر گیاه بودند. نتیجه گیری: این مطالعه نشان داد که مصرف پودر گیاه باعث کاهش شاخص های نامتناسب پلاسما (قند خون، تری گلیسیرید، کلسترول خون) و پراکسیداسیون لیپیدها (مالون دی آلدئید) در بیماران دیابتی می شود.

Title: بررسی میزان تأثیر برنامه آموزشی بر تحرک بدنی خانم‌های خانه دار متأهل 45-20 ساله شهر نور

http://tbj.ssu.ac.ir/article-1-2487-fa.html

چکیده مقدمه: انجام فعالیت جسمانی منظم به‌عنوان اولین اولویت سبک زندگی سالم عنوان‌شده است. هدف این مطالعه بررسی تأثیر آموزش بر تحرک بدنی خانم‌های خانه‌دار بود. روش بررسی: تعداد 106 نفر در قالب دو گروه (53 نفر گروه مداخله و 53 نفر گروه کنترل) با روش نمونه‌گیری تصادفی انتخاب شدند. برای اندازه­گیری از پرسشنامه بین‌المللی فعالیت فیزیکی استفاده شد. پرسشنامه‌ها به‌صورت پیش‌آزمون و پس‌آزمون در دو گروه مداخله و کنترل با فاصله 3 ماه و بعد از آموزش در گروه مداخله تکمیل گردید. آموزش به صورت سخنرانی، پاورپوینت، سی دی آموزشی و پمفلت مرتبط با موضوع، در اختیار گروه مداخله قرار گرفت. داده­ها با استفاده از آزمون­های ضریب همبستگی پیرسون، من­ویتنی، کروسکال والیس و ویلکاکسون تجزیه ‌و تحلیل شد. یافته‌ها: نتایج نمره حیطه تفریح، ورزش و فعالیت اوقات فراغت در گروه مداخله تفاوت معنی­ داری نشان داد. همچنین در معادل پیاده ­روی کلی گروه مداخله تفاوت معنی­ داری مشاهده شد. نتیجه‌گیری: یافته ها نشان می دهد  آموزش با استفاده از سی دی و پمفلت در کنار آموزش به وسیله پاورپوینت و وسایل کمک آموزشی، و استمرار آن می­ تواند موجب افزایش فعالیت­های ورزشی و تحرک بدنی  زنان خانه دار شود. این مقاله بر گرفته از پایان نامه کارشناسی ارشد دانشکده بهداشت، دانشگاه علوم پزشکی و خدمات بهداشتی درمانی شهید صدوقی یزد می باشد.

Title: کاربرد فرایند تلفیقی انعقاد و لخته سازی توام با تجزیه فتوکاتالیستی (TiO2/UV- A ) در حذف رنگ 2- ( متوکسی کربونیل آمینو- متیل) – آکریلیک اسید متیل استر از فاضلاب سنتتیک

http://tbj.ssu.ac.ir/article-1-1851-fa.html

مقدمه: پسابهای حاوی رنگ به دلیل اثرات سوء بهداشتی و زیست محیطی برای سلامت عمومی خطرناک هستند. هدف از این مطالعه کاربرد  فرایند تلفیقی  انعقاد و لخته سازی توام با تجزیه فتوکاتالیستی در حذف رنگ  2- ( متوکسی کربونیل آمینو- متیل) – آکریلیک اسید متیل استر از فاضلاب سنتتیک است. روش بررسی: در این تحقیق حذف رنگ مذکور به کمک مواد منعقد کننده و فرایند فتوکاتالیستی در راکتورهای ناپیوسته انجام شد. پارامترهای غلظت رنگ، غلظت نانوذرات اکسید تیتانیوم، شدت پرتو، زمان تماس، pH و COD مورد بررسی قرار گرفت. غلظت رنگ باقیمانده با استفاده از دستگاه اسپکتروفتومتر تعیین شد. یافته ها:نتایج این تحقیق نشان داد که حذف رنگ و COD توسط فرایند انعقاد و لخته سازی در شرایط بهینه به ترتیب  72 و5/58 درصد بدست آمد.و متعاقبا با استفاده از فرایند فتوکاتالیستی(TiO2/UV- A ) بیشترین درصد حذف رنگ و COD در غلظت 150 میلی گرم در لیتر نانو ذره و با شدت اشعه 480 میکرو وات بر سانتی متر مربع به ترتیب 98 و 8/90 درصد در طی زمان تماس 75 دقیقه و pH برابر 6 گزارش شد. نتیجه گیری: با توجه به نتایج بدست آمده، کاربرد فرایند تلفیقی  انعقاد و لخته سازی توام با تجزیه فتوکاتالیستی(TiO2/UV- A ) در حذف رنگ 2- ( متوکسی کربونیل آمینو- متیل) – آکریلیک اسید متیل استر و COD تحت شرایط بهینه بدست آمده بسیار موثر بوده و میتوان از آن در تصفیه فاضلاب صنعت خودروسازی استفاده نمود

Title: بررسی استفاده مجدد از پساب شهرکهای صنعتی جهت مصارف کشاورزی و آبیاری(مطالعه موردی: تصفیه خانه شهرک صنعتی جهان آباد میبد)

http://tbj.ssu.ac.ir/article-1-676-fa.html

مقدمه: استان صنعتی یزدبا دارا بودن شهرکهای صنعتی متعدد در محدوده خود و به دنبال آن حجم فاضلابهای تولیدی ناشی از این شهرکها با مشکل کم آبی بشدت مواجه است. بنابراین لزوم تصفیه مناسب فاضلابها و مدیریت کارآمد پسابهای تولیدی شهرکهای صنعتی این استان به طور جدی می بایست مطرح و مورد پیگیری مسئولانقرار گیرد. هدف از این مطالعه امکان سنجی استفاده مجدد از پساب شهرک صنعتی جهان آباد میبد جهت مصارف کشاورزی و آبیاری می باشد. روش بررسی: مطالعه حاضر یک پژوهش توصیفی مقطعی است. در این تحقیق، از مقادیر متوسط حاصل از نتایج نمونه برداری و آزمایشات انجام گرفته بر روی پساب خروجی تصفیه خانه شهرک صنعتی جهان آباد میبد در سال1391(مرداد ماه لغایت دی ماه) استفاده شده است. مقادیر متوسط پارامترهای BOD5،COD، TSSو ... با استانداردهای تعیین شده توسط سازمان حفاظت محیط زیست مقایسه و در نرم افزار اکسل تحلیل گردید. یافته ها: طبق نتایج بدست آمده مقادیر متوسط پارامترهای کیفیCOD،BOD5 ، TSS ، SO4،pH وکدورت مورد بررسی از پساب خروجی تصفیه خانه به ترتیب312،160، 92،162(mg/L)، 2/8 و 4/31(NTU)تعیین گردید. نتیجه گیری: نتایج تحقیق نشان می­دهد مقادیر متوسط پارامترهای کیفی مورد بررسی از پساب خروجی تصفیه خانه مورد مطالعه به غیر از  BOD5وCOD در محدوده استاندارد و حد مجاز جهت مصارف کشاورزی و آبیاری قرار دارد که ممکن است عملکرد زیست محیطی نامطلوب این دو پارامتر را در پی داشته باشد.

Title: بررسی اثر باریکه الکترونی بر کیفیت شیمیایی و خواص حسی گوشت شترمرغ

http://tbj.ssu.ac.ir/article-1-2082-fa.html

مقدمه: پرتودهی یکی از استراتژی‌های مداخله‌گر به‌منظور کنترل مؤثر میکروارگانیسم‌های پاتوژن منتقله از غذا می‌باشد که باعث کاهش بار میکروبی و افزایش مدت‌زمان نگهداری گوشت می‌شود. روش بررسی: در این مطالعه نمونه‌های گوشت شترمرغ با باریکه الکترونی به میزان 1.5، 3 و 5 کیلوگری مورد تیمار قرارگرفته و در دمای C°1±4 به مدت 15 روز نگهداری شدند. پس از پرتودهی در بازه‌های زمانی 5 روزه، به مدت 15 روز تغییرات شیمیایی و ارگانولپتیکی گوشت مورد ارزیابی قرار گرفت. همچنین میزان کل نیتروژن آزاد و اسید تیوباربیتوریک برای بررسی تغییرات شیمیایی اندازه‌گیری گردید. یافته ها: پرتودهی به‌طور معنی‌داری باعث کاهش میزان کل نیتروژن آزاد در نمونه‌های پرتودیده شد. همچنین افزایش میزان اسید تیوباربیتوریک نیز در نمونه‌های پرتودیده شده معنی‌دار بود که با دور پرتودهی و طول مدت نگهداری رابطه مستقیم داشت و علی‌رغم افزایش میزان اسید تیوباربیتوریک، پرتودهی اثر معنی‌داری بر خواص حسی گوشت شترمرغ نداشت. نتیجه گیری: نتایج این مطالعه نشان می‌دهد که پرتودهی گوشت شترمرغ با باریکه الکترونی باعث افزایش مدت‌ زمان نگهداری گوشت شترمرغ به مدت حداقل دو هفته در دمای یخچال بدون تغییر محسوس در خواص حسی این گوشت می‌شود.

Title: بررسی تعیین کننده های رفتارهای محافظت کننده از سرطان ریه در کارگران شرکت ذوب آهن اصفهان بر اساس تئوری انگیزش محافظت در سال 1392

http://tbj.ssu.ac.ir/article-1-1226-fa.html

مقدمه: سرطان ریه به عنوان سردسته ی علتهای مرگ در میان انواع سرطانها شناخته می شود. از آنجا که در کشور ما در خصوص تعیین کننده های رفتار های محافظت کننده از سرطان ریه در میان کارگران، اطلاعات علمی جامعی در دسترس نیست، طی مطالعه ای، تعیین کننده های رفتارهای محافظت کننده از سرطان ریه در کارگران شرکت ذوب اهن اصفهان بر اساس تئوری انگیزش محافظت بررسی شد. روش بررسی: این مطالعه توصیفی-مقطعی بر روی 253 نفر از کارگران کارخانه ی ذوب اهن اصفهان که به طور تصادفی انتخاب شدند، انجام شد. در این پژوهش از پرسشنامه ی محقق ساخته برای سنجش تعیین کننده های رفتارهای محافظت کننده از سرطان ریه استفاده شد. اطلاعات جمع آوری شده  با استفاده از آزمونهای آماری T-Test، ANOVA و ضریب همبستگی مورد تجزیه و تحلیل قرار گرفت. یافته ها: از افراد مورد مطالعه 2/54% همیشه معاینات پزشکی دوره ای را برای اطمینان از وضعیت سلامتی ریه هایشان انجام می دادند به علاوه 7/27% افراد برای حفظ سلامت ریه های خود از مواد کم خطر به جای مواد خطرناک استفاده می کردند. از بین سازه های تئوری انگیزش محافظت، سازه ی شدت درک شده بیشترین میانگین نمره(83/5 ±33/41) و سازه ی ترس کمترین میانگین نمره(49/2 ±77/9) را داشت. کل سازه های تئوری انگیزش محافظت، 6/22%  واریانس رفتار های محافظت کننده از سرطان ریه را پیش بینی نمودند. نتیجه گیری: با توجه به  اثر بخشی تئوری انگیزش محافظت درشناخت تعیین کننده های رفتارهای محافظت کننده از سرطان ریه،  می توان از این تئوری به عنوان چارچوبی در طراحی برنامه های آموزشی به منظور افزایش انگیزش کارگران جهت اتخاذ رفتار های محافظت کننده از سرطان ریه استفاده کرد.

Title: تاثیرتوسعه بانکی بر انتشار CO2 درایران

http://tbj.ssu.ac.ir/article-1-1483-fa.html

مقدمه: شناسایی تاثیر مصرف انرژی، رشد اقتصادی و تجارت خارجی بر میزان انتشار  CO2تا کنون هم در بعد داخلی و هم در بعد بین الملل در سطح وسیعی بررسی شده است، هر چند در زمینه چگونگی تاثیرگذاری توسعه مالی و توسعه بانکی بر میزان انتشار CO2 تحقیقات بسیار اندکی بویژه در ایران صورت گرفته است. بنابراین هدف اصلی مقاله کنونی پوشش شکاف تحقیقاتی در این زمینه می باشد. روش بررسی: مطالعه حاضر یک تحلیل  سری زمانی طی سال های 1390- 1350 می باشد. جهت بررسی وجود یا عدم وجود رابطه بلندمدت همجمعی میان میزان انتشار  CO2و شاخص های توسعه بانکی در ایران، ضرایب بلند مدت با بهره گیری از روش های خود بازگشتی با وقفه های توزیعی(ARDL) و حداقل مربعات پویا (DOLS ) برآورد گردید. همچنین جهت اجرای آزمون های اقتصاد سنجی از نرم افزارهای Microfit(5.0) و Eviews(9.0) استفاده شد. یافته ها: نتایج نشان داد نسبت مانده تسهیلات سیستم بانکی به بخش خصوصی به تولید ناخالص داخلی(شاخص توسعه بانکی) موجب کاهش انتشار CO2 در ایران می گردد. همچنین نتایج منطبق بر تئوری ها و بسیاری از مطالعات تجربی نشان داد تاثیر مصرف انرژی بر انتشار CO2در بلند مدت مثبت و معنادار است. نتیجه گیری: دولت می تواند با سیاست گذاری منسجم روی پرداخت تسهیلات سیستم بانکی به شرکت ها، کیفیت محیط زیست را ارتقاء دهد. زیرا در این چارچوب شرکت ها برای دریافت تسهیلات از سیستم بانکی ملزم به اجرای برنامه های مدون برای کاهش پیامدهای منفی مصرف انرژی شده و این در بلند مدت منجر به انتشار  CO2 کمتر می شود

Title: دیدگاه ها و نظرات مادران درباره آموزش مسایل جنسی به دختران نوجوان خود:یک مطالعه کیفی

http://tbj.ssu.ac.ir/article-1-1736-fa.html

مقدمه:مادران در انتقال اطلاعات و آموزش رفتارهای سالم از جمله مسایل جنسی، به دختران خود نقش کلیدی دارند. اطلاع از دیدگاه‌ها و نظرات مادران، پیش نیاز اصلی در طراحی مداخلات اثربخش برای توانمندسازی آنان است تا بتوانند به عنوان واسطه های آموزشی در این حوزه، نقش آفرینی کنند. این مطالعه با هدف استخراج نظرات مادران درباره آموزش مسایل جنسی به دختران نوجوان خود در شهر ماهشهر انجام شده است. روش بررسی: این پژوهش کیفی پدیدارشناسی، از طریق مصاحبه عمیق و بحث گروهی متمرکز بامادران دارای دختر نوجوان 18-12 ساله انجام شد. مصاحبه‌ها با هفت مادر و جلسات بحث گروهی با حضور 28 مادردر پنج گروه انجام شد. محوراصلی بحث‌ها آموزش مسایل جنسی به دختران و نحوه انجام آن بود. نظرات ارائه شده به طور کامل ثبت شد و پس از دسته بندی، تم‌های اصلی استخراج و تحلیل شد. یافته‌ها:پنج موضوع اصلی حاصل از مطالعه شامل لزوم آموزش مسایل جنسی به دختران نوجوان، منابع اطلاعاتی مادران درباره مسایل جنسی در دوران نوجوانی خود، منابع آموزشی پیشنهادی، موانع ارایه این آموزش‌ها، لزوم توانمند سازی مادران برای آموزش مسایل جنسی و پیشنهادات برای تدوین برنامه آموزشی مادران بود. نتیجه گیری: بیشتر مخاطبان به لزوم آموزش مسایل جنسی به دختران نوجوان به شکل محدود اعتقاد داشتند،مادران آموزش دیده را بهترین فرد برای آموزش دختران می‌دانستند وموانعی مانند خجالت از طرح موضوع، نگرانی از گستاخی و کنجکاوی دختران، آگاهی ناکافی درباره مسایل جنسی و نبود مهارت برقراری ارتباط موثر با دختران خود را بیان کردند.

Title: بررسی شیوع و عوامل خطر ناهنجاری مادرزادی قلبی (مقاله مروری)

http://tbj.ssu.ac.ir/article-1-1269-fa.html

چکیده مقدمه :نقائص مادرزادی قلب به وضعیتی گفته می شود که از زمان تولد بوجود می آید و برروی ساختار قلب نوزاد و همچنین عملکرد آن تاثیر میگذارد،این نقائص میتواند نوع مختلف از خفیف (مثل یک سوراخ کوچک دربین حفره های قلب)تا نوع شدید(مثل نقص یا ضعف در قسمتی از شکل قلب)داشته باشد. روش بررسی:این مقاله یک مطالعه مروری است که در آن از مقالات منتشر شده به زبان فارسی و انگلیسی که در پایگاههای معتبریمثل  Pubmed,Springer ,SID  Medline ,Google Scholar, نمایه شده استفاده است،همچنین برای این بررسی از کلید واژه های مرتبط  و موجود در MESH استفاده و بدون محدودیت زمانی در پایگاههای قید شده جستجو شد. یافته ها:شیوع ناهنجاریبه طور کلی کمتر از یک مورد در صد تولد بدست آمد و در بین ناهنجاری ها نفص دیواره ی بطنی(VSD)،نقص دیواره ی دهلیزی(ASD) ،بازبودن مجرای شریانی(ASD)و تترالوژی فالوت(TOF)بیشترین فراوانی را در بین انواع ناهنجاری های مادرزادی قلب به خود اختصاص  دادند.عواملی چون سن بالای والدین هنگام لقاح،ابتلای مادران به دیابت ،آنفلوآنزا و بیماری های تب دار ومصرف دارو در طی بارداری و عدم مصرف مولتی ویتامین قبل و حین بار داری توسط مادران در تولد نوزاد با ناهنجاری مادرزادی قلب تاثیر داشتند. نتیجه گیری:با توجه به مطالعات انجام شده و وجود عوامل خطر متعدد در ایجاد این ناهنجاری لزوم یک برنامه ریزی دقیق جهت بررسی بیشتر در ارتباط با عوامل خطر ایجاد این ناهنجاری و همچنین مداخلات تاثیر گذار به منظور کاهش عوامل خطر شناسایی شده به خصوص در دوران بارداری ضروری به نظر می رسد. این مقاله برگرفته از پایان نامه کارشناسی ارشد بهداشت و ایمنی مواد غذایی، دانشکده بهداشت، دانشگاه علوم پزشکی و خدمات بهداشتی درمانی شهید صدوقی یزد می باشد.
